# Comprehensive Genetic Analysis of Tuberculosis and Identification of Candidate Biomarkers

**DOI:** 10.3389/fgene.2022.832739

**Published:** 2022-03-07

**Authors:** Zilu Wen, Liwei Wu, Lin Wang, Qinfang Ou, Hui Ma, Qihang Wu, Shulin Zhang, Yanzheng Song

**Affiliations:** ^1^ Department of Scientific Research, Shanghai Public Health Clinical Center, Fudan University, Shanghai, China; ^2^ Department of Thoracic Surgery, Shanghai Public Health Clinical Center, Fudan University, Shanghai, China; ^3^ Department of TB, The fifth people’s hospital of Wuxi, Wuxi, China; ^4^ TB Center, Shanghai Emerging and Re-emerging Infectious Diseases Institute, Shanghai, China

**Keywords:** tuberculosis, second generation sequencing, diagnosis, WGCNA, DEGs

## Abstract

**Purpose:** The purpose of this study is to use the data in the GEO database to analyze, screen biomarkers that can diagnose tuberculosis, and verification of candidate biomarkers.

**Materials and methods:** GSE158767 dataset were used to process WGCNA analysis, differential gene analysis, Gene ontology and KEGG analysis, protein-protein network analysis and hub genes analysis. Based on our previous study, the intersect between WGCNA and differential gene analysis could be used as candidate biomarkers. Then, the enzyme-linked immunosorbent assay was used to validate candidate biomarkers, and receiver operating characteristic was used to assess diagnose ability of candidate biomarkers.

**Results:** A total of 412 differential genes were screened. And we obtained 105 overlapping genes between DEGs and WGCNA. GO and KEGG analysis showed that most of the differential genes were significantly enriched in innate immunity. A total of 15 hub genes were screened, and four of them were verified by Enzyme-linked immunosorbent assay. CCL5 performed well in distinguishing the healthy group from the TB group (AUC = 0.723). And CCL19 performed well in distinguishing the TB group from the ORD groups (AUC = 0.811).

**Conclusion:** CCL19, C1Qb, CCL5 and HLA-DMB may play important role in tuberculosis, which indicated four genes may become effective biomarkers and could be conveniently used to facilitate the individual tuberculosis diagnosis in Chinese people.

## Introduction

Tuberculosis has been accompanied by human for thousands of years, and it is still a major public health problem that threatens human health. According to the World Health Organization tuberculosis report, about a quarter of the world’s population is infected with *M. tuberculosis* and thus at risk of developing TB disease (WHO, 2019). In 2018, about 10 million people were infected with TB. Meanwhile, there were 1.2 million (range, 1.1–1.3 million) TB deaths among HIV-negative people in 2018 (a 27% reduction from 1.7 million in 2000), and an additional 251,000 deaths (range, 223,000–281,000)3 among HIV- positive people (a 60% reduction from 620,000 in 2000). Geographically, eight countries accounted for two thirds of the global total: India (27%), China (9%), Indonesia (8%), the Philippines (6%), Pakistan (6%), Nigeria (4%), Bangladesh (4%) and South Africa (3%). Therefore, curbing the spread of tuberculosis is an urgent problem to be solved (WHO, 2019).

Tuberculosis is mainly transmitted by respiratory tract. Therefore, the early diagnosis of tuberculosis is very important. However, there are still some problems in the diagnosis of tuberculosis (Jakhar et al., 2020). For example, the gold standard sputum culture takes too long, and the false positive rate of tuberculosis antibody is too high (Chen J et al., 2020; Mansoori et al., 2020). According to the World Health Organization, 55% of lung cases were confirmed bacteriologically in 2018 (WHO, 2019). And we should increase the percentage of cases confirmed bacteriologically by scaling up the use of recommended diagnostics (e.g., rapid molecular tests) that are more sensitive than smear microscopy. The biomarkers produced by the immune reaction in the process of infection with *Mycobacterium tuberculosis* is a relatively accurate and rapid molecular test. Therefore, there is an urgent need to find novel biomarkers to solve the problem of tuberculosis diagnosis.

With the development of bioinformatics technology, more and more new techniques are applied to analyze expression profile data. Differential gene analysis is the most classical analysis method, which plays an important role in this field of biomarkers by a series of statistical algorithms to find differential genes between different subgroups (Liu et al., 2022; Zhao et al., 2022). WGCNA (weighted gene co-expression network analysis) is a topological network that can establish the linkage between gene modules and clinical traits, and the genes classified into the same module are all linked to selected clinical traits, which can then be used for subsequent analysis and experiments (Nguyen et al., 2021; Ye et al., 2021). The combination of differential gene analysis with WGCNA allows the screening of genes that are differentially expressed and associated with selected clinical traits, which can be used for further screening to identify the final biomarkers.

In this study, we used the data from the GEO database to conduct a comprehensive bioinformatics analysis to screen out possible biomarkers for the diagnosis of tuberculosis. Sequencing data from lung tissues were used to analyze and biomarker were screened for lung tissue of high metabolic activity and lung tissue of low metabolic activity, which can reveal specific features of host lung immunity, and can screen for biomarkers associated with immune and metabolic activity, thus improving the sensitivity and specificity of the biomarkers. Enzyme-linked immunosorbent assay (ELISA) was used to validate the screened biomarkers. The results of the verification are used for statistical analysis and the establishment of predictive models to help doctors diagnose tuberculosis.

## Materials and Methods

### Acquisition of RNA Data

The gene expression data of tuberculosis patients were obtained from GEO database (Barrett et al., 2013). The selection criteria for the GEO database were set as follows: tissue; RNA high-throughput sequencing; release time descending order. Finally, GSE158767 was selected as a candidate dataset. Gene expression data of 10 lung tissue samples were obtained from GSE158767, of which 5 were metabolic high and five were metabolic low. According to the description of GSE158767, standard uptake values (SUV) of PET-CT greater than three were considered metabolic high, while SUV less than three were considered metabolic low. In addition, the platform of GSE158767 was Illumina NovaSeq 6,000 (GPL24676). According the guideline of edgeR package, gene with low read counts cannot be used for further analysis. Therefore, the gene with cpm (count per million) ≥ 1 was kept in this study. All the data obtained have been normalized for further analysis. We used the function rpkm in edgeR package to conduct normalization process.

### WGCNA Analysis

Co-expression networks have facilitated the development of network-based gene screening methods that can be used to identify candidate biomarkers and therapeutic targets. In this study, we constructed a gene expression data map of GSE158767 to construct a gene expression network based on the WGCNApackage (Zuo et al., 2018). WGCNA was used to identify the genes which were related with clinical phenotype. In order to build the scale-free network, we used the function pickSoftThreshold to select soft powers *β* = 3 (Liang et al., 2020; Zhao et al., 2021). Then, we used the following formula to create the adjacency matrix:

aij=|Sij|β 

Sij: similarity matrix which is done by Pearson correlation of all gene pairs

β:softpower value

And then the adjacency matrix was transformed into a topological overlap matrix (TOM) as well as the corresponding dissimilarity (1-TOM). Then, a hierarchical clustering tree diagram of the 1-TOM matrix was constructed to classify similar gene expression into different gene co-expression modules (Xu M et al., 2020). To further identify functional modules in the co-expression network, module-trait associations between modules and clinical feature information were calculated based on previous studies. As a result, modules with high correlation coefficients were considered as candidates for correlation with clinical features and were selected for subsequent analysis.

### Screening DEGs and Intersect Between DEGs and Interesting Module

The gene expression data matrix was uploaded to NetworkAnalyst (https://www.networkanalyst.ca) for further analysis (Zhou G et al., 2019). The criteria for filtering DEGs are as follows:∣logFC∣>1, adjust *p*-value <0.05 (Bos et al., 2017). The *p*-value was adjusted by the Benjamini–Hochberg method to control for the false discovery Rate (FDR). The DEGs were visualized as volcano plot by using R package ggplot2. Then, the intersect between DEGs and co-expression genes that were extracted from the interesting module were used to identify potential biomarkers, which were visualized as a Venn diagram using the R package VennDiagram.

### Enrichment Analysis

The metascape database (https://metascape.org/gp) is a gene annotation and analysis database (Zhou Y et al., 2019). The Kyoto Encyclopedia of Genome and Genome (KEGG) is a database resource for understanding advanced functions and biological systems from large-scale molecular data generated by high-throughput experimental techniques (Kanehisa et al., 2017). Gene ontology (GO) analysis, including annotations of biological process (BP), molecular functional (MFS) and cellular module (CCS), is the main bioinformatics tool for annotating genes and analyzing the biological processes of these genes. We used metascape online database for bioinformatics analysis of overlapping genes. And a false discovery rate (FDR) of less than 0.05 were considered statistically significant (Bos et al., 2017). The results obtained are visualized by R-package ggplot2.

### Protein‐protein Interaction Network Construction and Module Analysis

We used the Search Tool for the Retrieval of Interacting Genes (STRING) database (http://string‐db.org) to obtain the Protein‐protein interaction (PPI) network (Szklarczyk et al., 2015). Import the network data into Cytoscape (Version: 3.8.0) for further analysis (Smoot et al., 2011). CytoHubba, a plug-in of Cytoscape, was used to filter hub genes (Chin et al., 2014). And 15 hub genes were screened out by MCC method and sequentially ordered. More forward rankings are represented by redder color. Taking the intersection of 15 hub genes and our previous research results (Wang et al., 2021), the obtained genes are used for experimental verification.

### Verification of the Candidate Biomarkers

Enzyme-linked immunosorbent assay (ELISA) is a qualitative and quantitative detection method that uses antigen-antibody specific binding for immune response. The experimental arrangement mainly involves the sandwich method and the competition method. Candidate biomarkers for TB were validated using the ELISA kits (USCN Life Sciences; Wuhan, China). Protein levels in plasma were detected according to the manufacturer instructions. Collect plasma using EDTA as an anticoagulant. Centrifuge samples for 15 min at 1,000×g at 2–8°C within 30 min of collection. Remove plasma and assay immediately or store samples in aliquot at -80 °C for later use. Avoid repeated freeze/thaw cycles. We measured protein levels of candidate biomarkers in plasma from 88 patients with pulmonary tuberculosis, 88 healthy controls and 88 ORDs (Other respiratory diseases). Student’s t-test was used to compare the differences between the two groups, and *p* < 0.05 was considered statistically significant. Graphpad Prism (version 8.0) was used to visualize the results as well as for statistical analysis. To determine the diagnostic efficacy of biomarkers, the R package pROC was used to perform receiver operating characteristic (ROC) curve.

## Results

**FIGURE 1:**
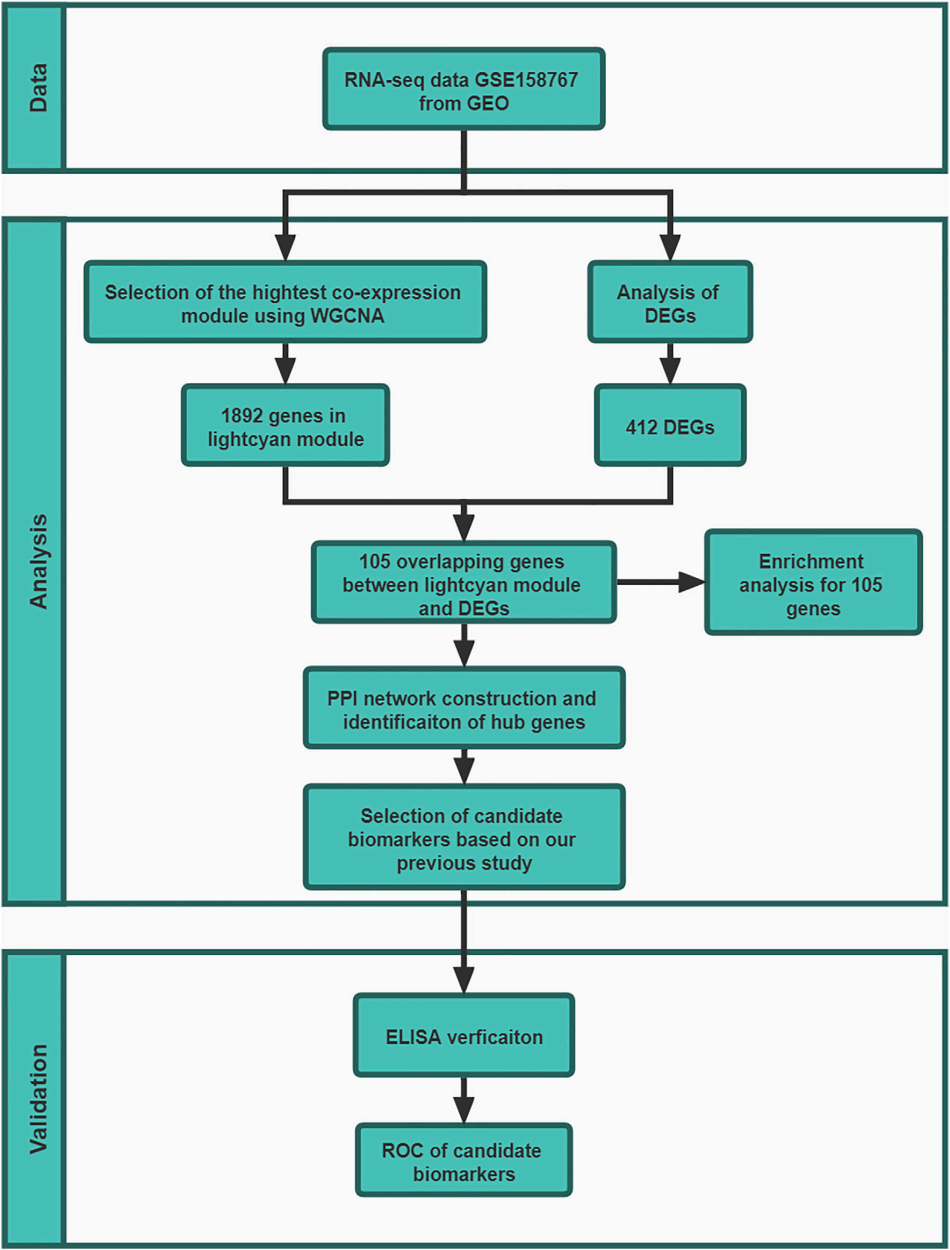
The work flow of this study. In GSE158767, five lung tissue samples from sputum-negative tuberculosis patients with high metabolic activity and 5 with low metabolic activity. At validation stage, 88 donors in HC group, 88 donors in TB groups and 88 donors in ORD groups.

The work flow of this study was shown in Figure 1.

### WGCNA Analysis and Interesting Modules

**FIGURE 2:**
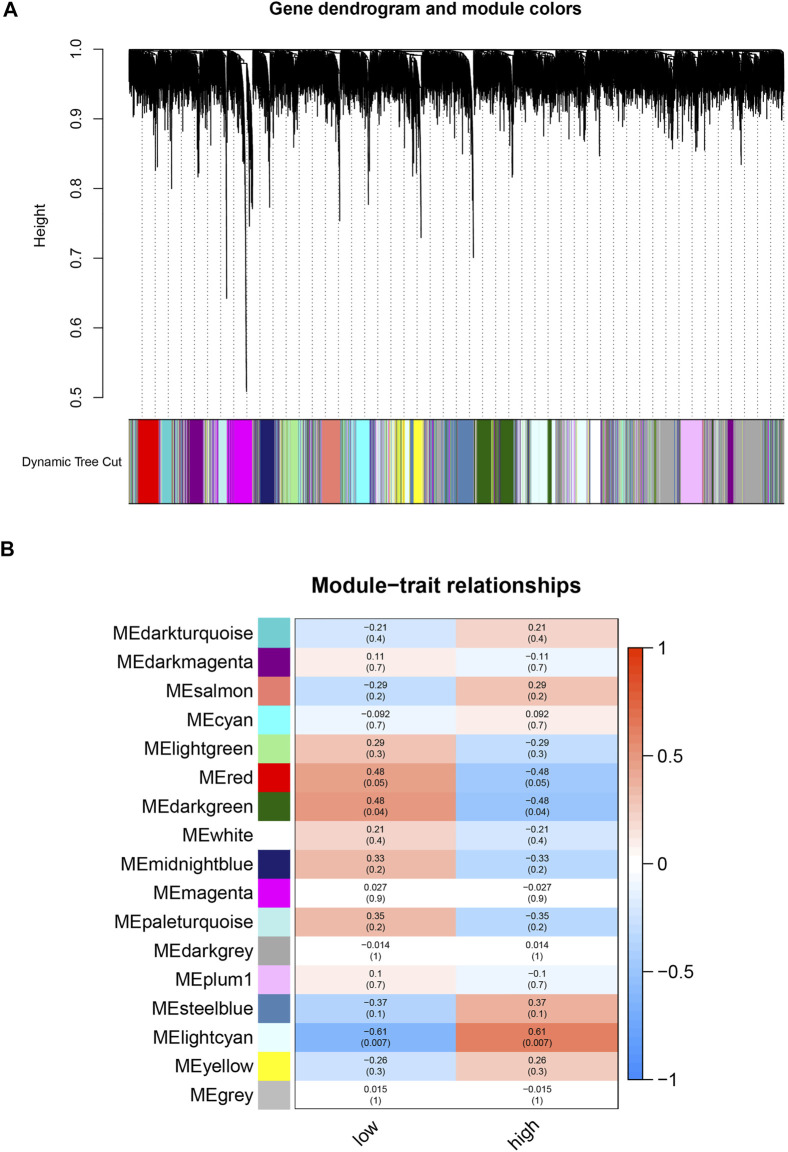
Identification of modules related with the clinical traits in the GSE158767. **(A)** The Cluster dendrogram of co-expression network modules was ordered by a hierarchical clustering of genes based on the 1-TOM matrix. Each module was assigned different colors. **(B)** Module-trait relationships. Each row corresponds to a color module and column corresponds to a clinical trait (low and high). Each block contains the correlation value and *p*-value.

To find biomarkers associated with focal metabolic activity in TB patients, we constructed a gene co-expression network using the WGCNA package. All genes were divided into different modules and each module was assigned a different color (Figure 2A). The correlation between each module and two clinical features was assessed by plotting a heat map of module-trait relationships. The results of the module-trait relationship were shown in Figure 2B, indicating that the lightcyan module in GSE158767 had the highest correlation with the metabolism-high tissue (r = 0.61, *p* = 0.007).

### Intersect Between the DEGs and Interesting Module

**FIGURE 3:**
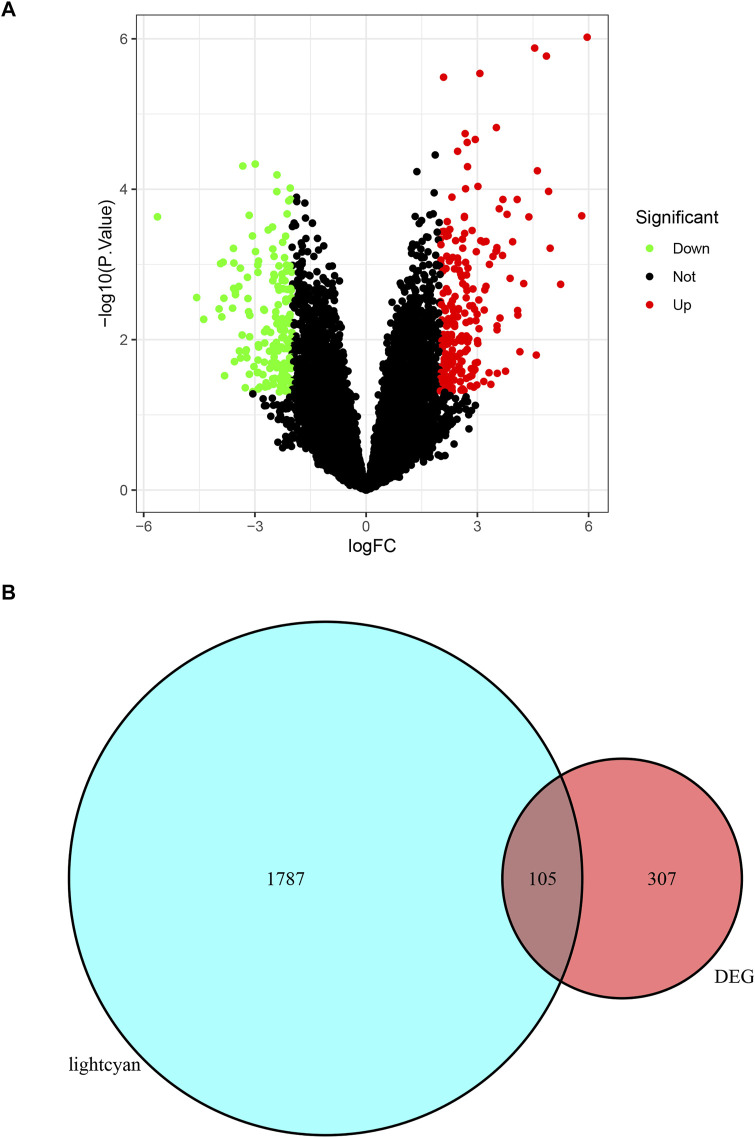
**(A)** The DEGs of GSE158767. The red dots present up-regulated genes, green dots present down-regulated genes, and black dots present none-regulated genes. **(B)** The venn diagram of intersect between DEGs and lightcyan module.

A total of 412 DEGs were identified by differential gene analysis (Figure 3A). The list of DEGs obtained was intersected with the genes in the lightcyan module and a total of 105 overlapping genes were identified (Figure 3B). These overlapping genes were dysregulated in expression in foci of increased metabolism and could be used as candidate biomarkers.

### Enrichment Analysis for Overlapping Genes

**FIGURE 4:**
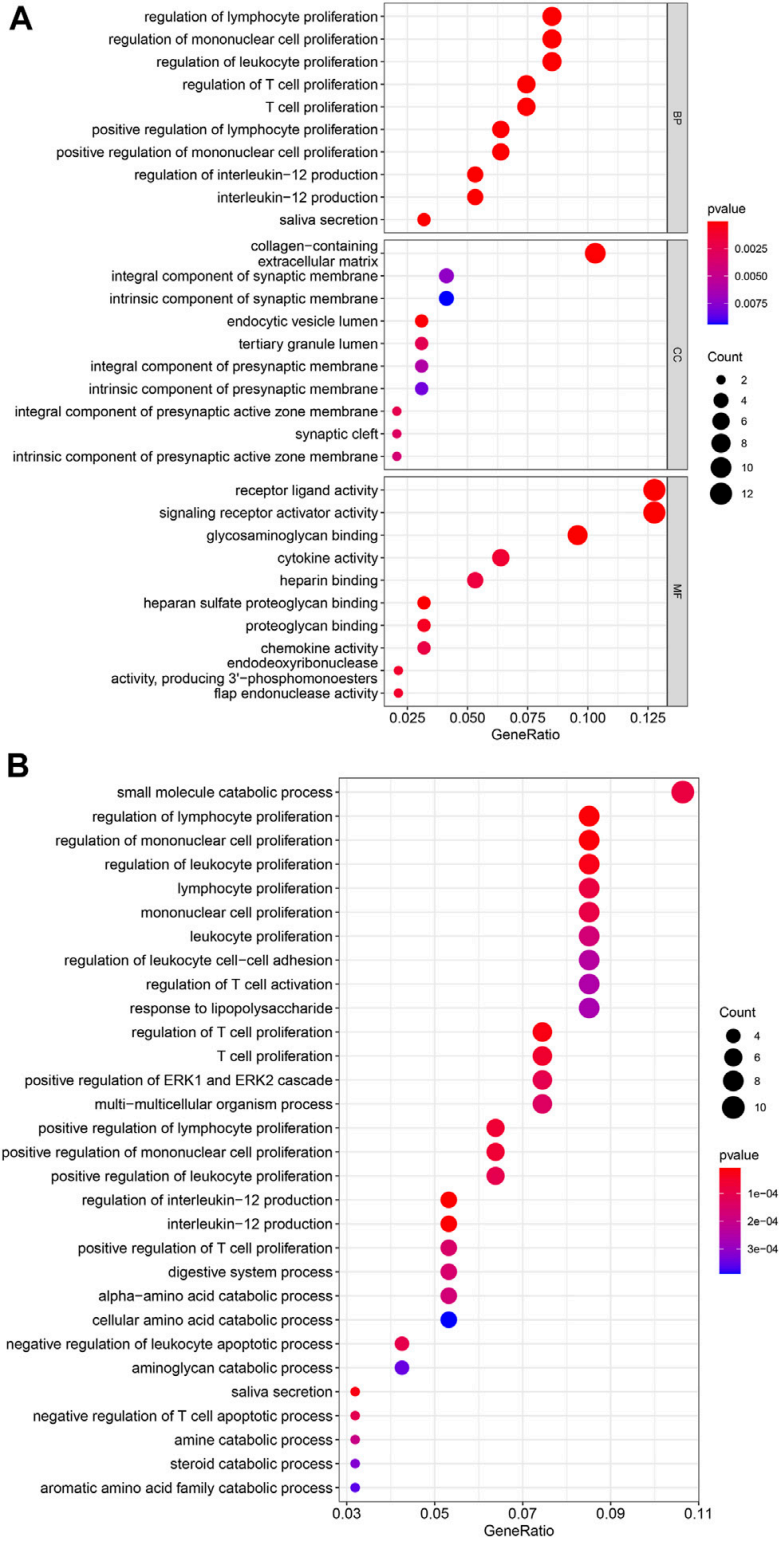
**(A)** GO enrichment analysis. BP, biological process; MF, molecular function; CC, cellular component. The colors present the *p*-value of each GO terms, and red present low while blue present high. **(B)** KEGG enrichment analysis. The colors present the *p*-value of each GO terms, and red present low while blue present high.

The results of GO enrichment analysis were shown in Figure 4A. In BP, the most genes were enriched in regulation of lymphocyte proliferation, regulation of mononuclear cell proliferation, and regulation of leukocyte proliferation. In CC, the most genes were enriched in collagen-containing extracellular matrix. In MF, the most genes were enriched in receptor ligand activity, and signaling receptor activator activity. The results of KEGG enrichment analysis were visualized as bubble plot (Figure 4B). The most genes were enriched in small molecule catabolic process, regulation of lymphocyte proliferation, regulation of mononuclear cell proliferation, regulation of leukocyte proliferation, and regulation of T cell proliferation.

### Protein-Protein Interaction Network and Hub Genes

**FIGURE 5:**
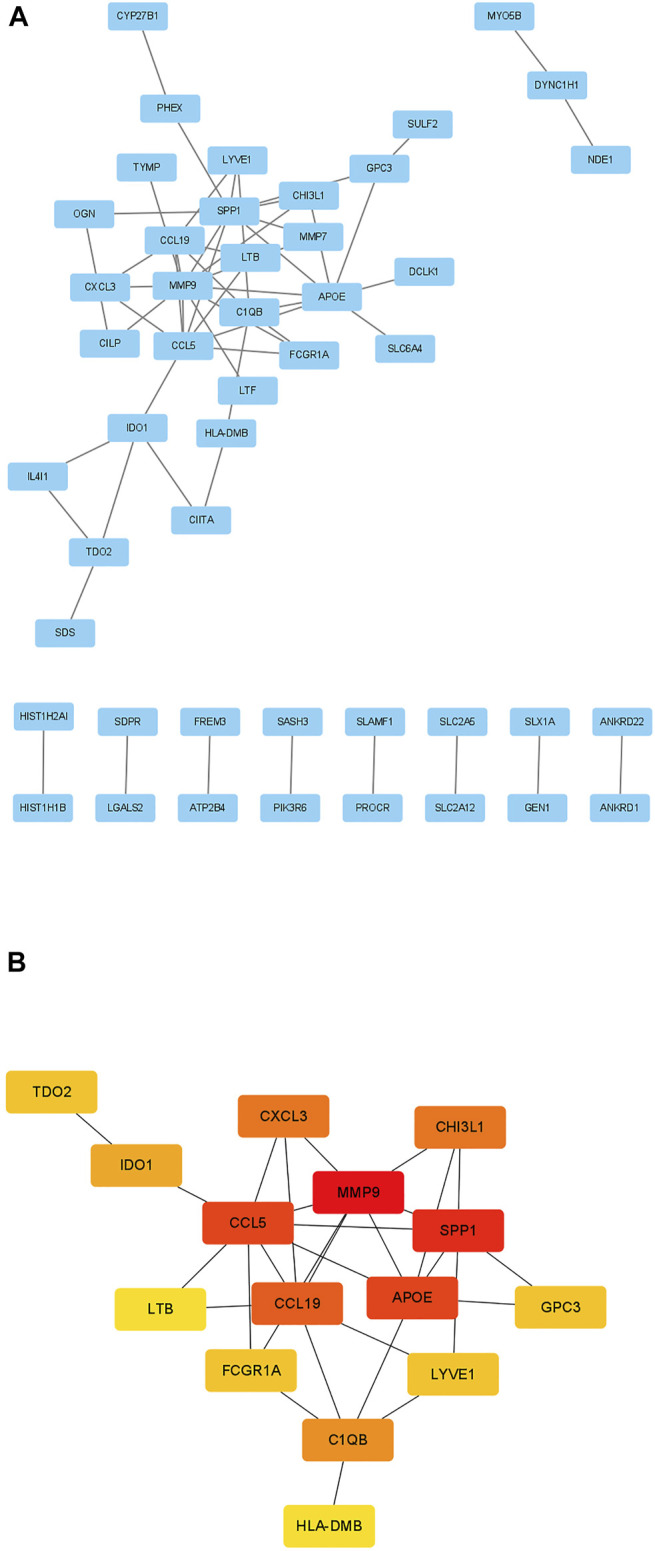
Identification of candidate biomarkers. **(A)** PPI network of 105 genes. **(B)** The 15 hub genes identified by MCC scores. The red presents high MCC scores while yellow presents low MCC scores.

The STRING database (https://www.string-db.org/) was used to construct PPI network among the overlapped genes (Figure 5A). The hub genes selected from the PPI network using the MCC of CytoHubba plugin were presented in Figure 5B. Based on MCC scores, the top 15 highest score genes were selected as hub genes (MMP9, CCL5, SPP1, CCL19, APOE, CXCL3, CHI3L1, IDO1, TDO2, GPC3, LTB, FCGR1A, LYVE1, C1QB, HLA-DMB). According to our previous study, we found CCL5, CCL19, C1QB and HLA-DMB were high expression in TB lung tissue, therefore we finally selected the above biomarkers for experimental verification (Wang et al., 2021).

### Experimental Verification

**FIGURE 6:**
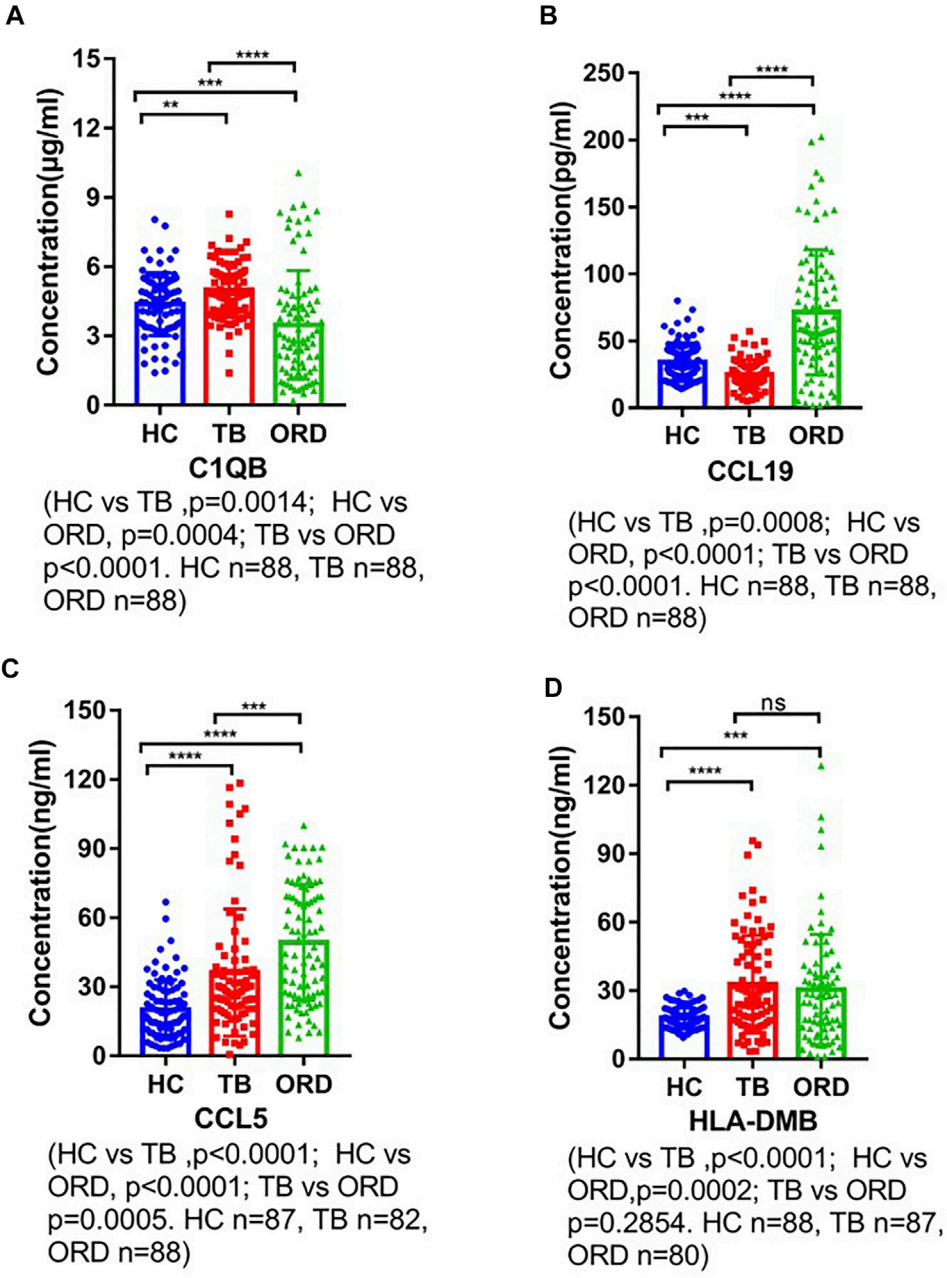
The ELISA verification of four biomarkers. HC, healthy control; TB, tuberculosis; ORD, other respiratory disease. **(A)** ELISA verification of C1QB. **(B)** ELISA verification of CCL19. **(C)** ELISA verification of CCL5. **(D)** ELISA verification of HLA-DMB.

**FIGURE 7:**
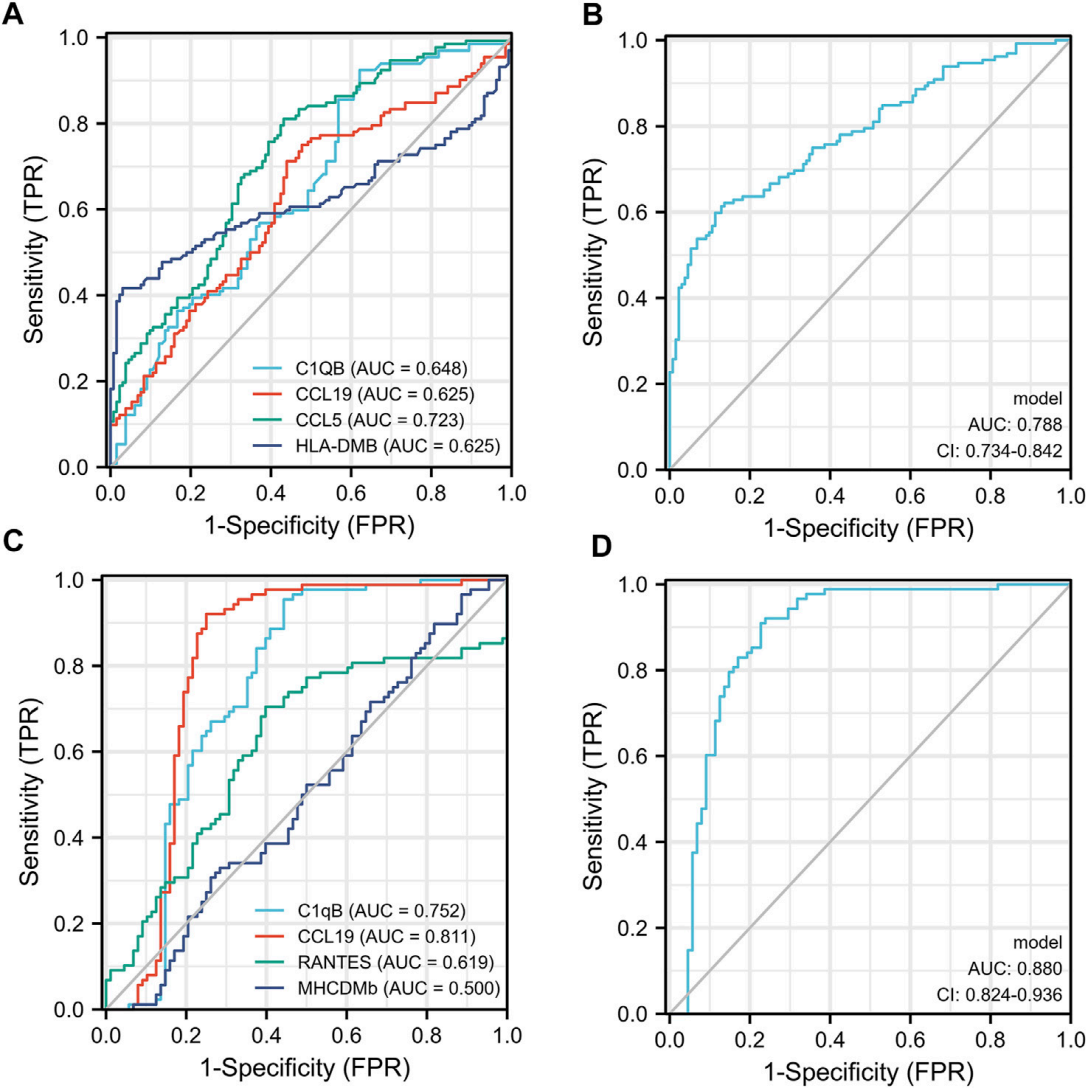
The ROC of four biomarkers. **(A)** Each biomarker plot one ROC (TB vs. HC). **(B)** Four biomarkers combined using logistic regression model (TB vs. HC). **(C)** Each biomarker plot one ROC (TB vs. ORD). **(D)** Four biomarkers combined using logistic regression model (TB vs. ORD).

We used ELISA to validate the four candidate biomarkers screened and the experimental results were shown in Figure 6. Among them, C1QB, CCL5 and CCL19 all showed good discriminatory properties, with statistically significant differences in the healthy, TB and ORD groups. While HLA-DMB was statistically significantly different in the healthy and TB groups, but not in the TB and ORD groups. We then assessed the diagnostic efficacy of the four markers using ROC, and the results were shown in Figure 7. CCL5 performed well in distinguishing the healthy group from the TB group (AUC = 0.723). And CCL19 performed well in distinguishing the TB group from the ORD groups (AUC = 0.811). After that, we used logistic regression analysis and the results specified that the four biomarkers performed better in combination for prediction (TB vs HC, AUC = 0.788; TB vs ORD, AUC = 0.880).

## Discussion

Tuberculosis is an infectious disease that can involve organs and tissues throughout the body and is caused by *Mycobacterium tuberculosis*. At present, TB remains the largest pathogenic cause of mortality worldwide, apart from COVID-19. Although the diagnostic criterion for tuberculosis is sputum positivity, the low rate of positivity by pathogenic methods has resulted in a large number of potential tuberculosis patients not being diagnosed in a timely manner, thus delaying treatment. Therefore, the development of biomarkers that can diagnose TB is demanded. The second-generation RNA sequencing technique was used to detect the specimens of tuberculosis patients taken out by operation. After that, the four candidate biomarkers were screened by bioinformatics. And then, using ELISA technology to analyze the plasma of tuberculosis group, health control group and ORD group, four biomarkers were verified. The results showed that four biomarkers were the most statistically significant. According to the results of ROC analysis, CCL19 may become a biomarker for the diagnosis of tuberculosis. And the diagnostic efficacy of the combination of these four biomarkers was also high according to the results of logistic regression analysis, but it still needs to be further confirmed by prospective studies.

The C1qB encodes the B-chain polypeptide of serum complement subcomponent C1q, which associates with C1r and C1s to yield the first component of the serum complement system (Bos et al., 2017). C1q is composed of 18 polypeptide chains which include 6 A-chains, 6 B-chains, and 6 C-chains. Each chain contains an N-terminal collagen-like region and a C-terminal C1q globular domain (Lubbers et al., 2020). Some previous studies (Chen et al., 2011; Radanova et al., 2012) demonstrated that c1q deficiency is associated with lupus erythematosus and glomerulonephritis. There is a certain discrepancy between this and our research results. Our results show that when the expression of C1qB is increased, it indicates that there may be inflammation caused by tuberculosis infection. This may be due to the different mechanisms of inflammation and autoimmune inflammation caused by *Mycobacterium tuberculosis* infection. The specific mechanism of C1qB in tuberculosis needs to be further studied.

CCL5 is a chemokine that participates in immune regulation and inflammation (Singh et al., 2020), and has chemotaxis to monocytes, memory T helper cells and eosinophils (Tavares et al., 2020). It has been widely studied in HIV infection. It mainly participates in cellular immune response and plays an important role in CD8+T cells. (Chen Y.-C et al., 2020). Some studies (Lee et al., 2019; Fujimoto et al., 2020; Yu-Ju Wu et al., 2020) have pointed out that CCL5 is a key target for the treatment of AIDS, tumors and even inflammation. In these results, the expression of CCL5 is increased, which is consistent with our study. We confirmed that the expression of CCL5 is also increased in inflammation caused by *Mycobacterium tuberculosis* infection. Combined with the results of other studies, CCL5 is likely to become a new target for the treatment of tuberculosis.

CCL19 is one of several CC cytokine genes clustered on the p-arm of chromosome 9. Cytokines are a family of secreted proteins involved in immunoregulatory and inflammatory processes (Yan et al., 2019). The CC cytokines are proteins characterized by two adjacent cysteines. The cytokine encoded by this gene may play a role in normal lymphocyte recirculation and homing. It also plays an important role in trafficking of T cells in thymus, and in T cell and B cell migration to secondary lymphoid organs (Saxena et al., 2019). It specifically binds to chemokine receptor CCR7 (Wang et al., 2019). Some studies (Yan et al., 2019; Xu H et al., 2020) have shown that the increased expression of CCL19 is related to virus infection. This is consistent with our research results. The host immunity of tuberculosis is mainly T cell immunity. However, there has not been any research on the CCL19 of tuberculosis. CCL19 may become a new target for tuberculosis treatment, and its diagnostic value has been confirmed by our research.

Since 2008 to date, more than nine thousand studies of TB biomarkers exist, and more than five thousand of them are diagnostic biomarkers. However, most of the diagnostic biomarkers have not been subsequently validated with large-scale data, and there are currently four biomarkers that have been validated with large-scale data (Zimmer et al., 2021). These four biomarkers are CRP, IL-6, IP-10, and TNF-α. CRP was the first to appear and is now in clinical use as an adjunct to the diagnosis of TB and to determine the efficacy of anti-TB therapy (Fusani et al., 2021). IL-6 and IP-10 have considerable potential to diagnose tuberculosis, and biologic companies already exist to develop related products (Zimmer et al., 2021). TNF-α is similar to above three biomarkers and can diagnose tuberculosis, showing a high diagnostic efficacy (Zimmer et al., 2021; Huang et al., 2022). However, the above biomarkers are still lacking in terms of diagnostic specificity, and no biomarkers with high specificity for TB can be identified yet, which may require more in-depth studies targeting host immunity.

In summary, the diagnostic efficacy of each biomarker alone is not satisfactory, but the diagnostic model formed by the combination of the four biomarkers is very effective. The diagnostic model had an AUC of 0.788 in distinguishing TB from HC and an AUC of 0.880 in distinguishing TB from ORD. In the field of diagnostic biomarkers, diagnostic models formed by the combination of several biomarkers are becoming more dominant because they can combine the advantages of each biomarker (Deng et al., 2022; Huang et al., 2022). In this study, new diagnostic biomarkers for tuberculosis were identified in blood using expression profile data obtained from lung tissue. Prior to validation at the plasma protein level, we also performed validation at the RNA level, but were unable to graph the four biomarkers due to their low RNA expression in whole blood. This further suggests that these four biomarkers are released into the blood after being synthesized as proteins in the lung tissue. This study had some limitations, firstly, only sequencing data from lung tissue were used in the screening stage, so it was difficult to state that the screened biomarkers were specific for TB. Secondly, the small number of samples sequenced made it prone to statistical bias. Further research of the four biomarkers and other biomarkers which were found by previous studies were needed to illustrate their diagnostic efficacy.

At present, the diagnosis of tuberculosis is still a major problem to be solved. We have developed an effective diagnostic tool to help clinicians identify TB patients early. In addition, it can also be used as a tool to judge the therapeutic effect of tuberculosis. In addition, the determination of the plasma content of the four biomarkers is a simple detection method. It can reduce the difficulty of tuberculosis diagnosis and reduce the economic burden of patients. These four biomarkers can even be made into kits to promote the use of tuberculosis, so as to diagnose tuberculosis as soon as possible.

## Conclusion

The study developed four novel biomarkers (CCL5, C1Qb, CCL19 and HLA-DMB) for diagnose of TB. Through the early diagnosis of tuberculosis, clinicians and patients can take more necessary measures in terms of treatment and follow-up.

## Data Availability

The original contributions presented in the study are included in the article/supplementary material further inquiries can be directed to the corresponding author/s.

## References

[B1] Barrett T. Wilhite S. E. Ledoux P. Evangelista C. Kim I. F. Tomashevsky M. (2013). NCBI GEO: Archive for Functional Genomics Data Sets-Update. Nucleic Acids Res. 41, D991–D995. 10.1093/nar/gks1193 23193258 PMC3531084

[B2] Bos S. Phillips M. Watts G. F. Verhoeven A. J. M. Sijbrands E. J. G. Ward N. C. (2017). Novel Protein Biomarkers Associated with Coronary Artery Disease in Statin-Treated Patients with Familial Hypercholesterolemia. J. Clin. Lipidol. 11 (3), 682–693. 10.1016/j.jacl.2017.03.014 28434814

[B3] Chen G. Tan C. S. Teh B. K. Lu J. (2011). Molecular Mechanisms for Synchronized Transcription of Three Complement C1q Subunit Genes in Dendritic Cells and Macrophages. J. Biol. Chem. 286 (40), 34941–34950. 10.1074/jbc.M111.286427 21862594 PMC3186423

[B4] Chen J. Han Y. S. Yi W. J. Huang H. Li Z. B. Shi L. Y. (2020). Serum sCD14, PGLYRP2 and FGA as Potential Biomarkers for Multidrug‐Resistant Tuberculosis Based on Data‐Independent Acquisition and Targeted Proteomics. J. Cel. Mol. Med. 24, 12537–12549. 10.1111/jcmm.15796 PMC7686995 32967043

[B5] Chen Y.-C. Chen S.-P. Li J.-Y. Chen P.-C. Lee Y.-Z. Li K.-M. (2020). Integrative Model to Coordinate the Oligomerization and Aggregation Mechanisms of CCL5. J. Mol. Biol. 432 (4), 1143–1157. 10.1016/j.jmb.2019.12.049 31931012

[B6] Chin C.-H. Chen S.-H. Wu H.-H. Ho C.-W. Ko M.-T. Lin C.-Y. (2014). cytoHubba: Identifying Hub Objects and Sub-Networks from Complex Interactome. BMC Syst. Biol. 8, S11. 10.1186/1752-0509-8-s4-s11 25521941 PMC4290687

[B7] Deng H. Li J. Ali Shah A. Lin G. Chen H. Ouyang W. (2022). Commonly Expressed Key Transcriptomic Profiles of Sepsis in the Human Circulation and Brain via Integrated Analysis. Int. immunopharmacology 104, 108518. 10.1016/j.intimp.2022.108518 35032827

[B8] Fujimoto Y. Inoue N. Morimoto K. Watanabe T. Hirota S. Imamura M. (2020). Significant Association between High Serum CCL5 Levels and Better Disease‐Free Survival of Patients with Early Breast Cancer. Cancer Sci. 111 (1), 209–218. 10.1111/cas.14234 31724785 PMC6942441

[B9] Fusani L. Tersigni C. Chiappini E. Venturini E. Galli L. (2021). Old Biomarkers in Tuberculosis Management: Are They Still Useful? a Systematic Review. Expert Rev. Anti-Infective Ther. 19, 1191–1203. 10.1080/14787210.2021.1898945 33722116

[B10] Huang W.-C. Lin H.-C. Yang Y.-H. Hsu C.-W. Chen N.-C. Tsai W.-C. (2022). Neutrophil-to-lymphocyte Ratio and Monocyte-To-Lymphocyte Ratio Are Associated with a 2-year Relapse in Patients with Multiple Sclerosis. Mult. Scler. Relat. Disord. 58, 103514. 10.1016/j.msard.2022.103514 35032880

[B11] Jakhar S. Bitzer A. A. Stromberg L. R. Mukundan H. (2020). Pediatric Tuberculosis: The Impact of "Omics" on Diagnostics Development. Int. J. Mol. Sci. 21 (19), 6979. 10.3390/ijms21196979 PMC7582311 32977381

[B12] Kanehisa M. Furumichi M. Tanabe M. Sato Y. Morishima K. (2017). KEGG: New Perspectives on Genomes, Pathways, Diseases and Drugs. Nucleic Acids Res. 45, D353–D361. 10.1093/nar/gkw1092 27899662 PMC5210567

[B13] Lee C. P. Nithiyanantham S. Hsu H. T. Yeh K. T. Kuo T. M. Ko Y. C. (2019). ALPK1 Regulates Streptozotocin‐induced Nephropathy through CCL2 and CCL5 Expressions. J. Cel Mol Med 23 (11), 7699–7708. 10.1111/jcmm.14643 PMC6815771 31557402

[B14] Liang W. Sun F. Zhao Y. Shan L. Lou H. (2020). Identification of Susceptibility Modules and Genes for Cardiovascular Disease in Diabetic Patients Using WGCNA Analysis. J. Diabetes Res. 2020, 4178639. 10.1155/2020/4178639 32455133 PMC7238331

[B16] Liu S.-L. Sun X.-S. Chen Q.-Y. Liu Z.-X. Bian L.-J. Yuan L. (2022). Development and Validation of a Transcriptomics-Based Gene Signature to Predict Distant Metastasis and Guide Induction Chemotherapy in Locoregionally Advanced Nasopharyngeal Carcinoma. Eur. J. Cancer 163, 26–34. 10.1016/j.ejca.2021.12.017 35032814

[B17] Lubbers R. van Schaarenburg R. A. Kwekkeboom J. C. Levarht E. W. N. Bakker A. M. Mahdad R. (2020). Complement Component C1q Is Produced by Isolated Articular Chondrocytes. Osteoarthritis and cartilage 28 (5), 675–684. 10.1016/j.joca.2019.09.007 31634584

[B18] Mansoori N. Pahlavanzadeh B. Arabmofrad F. (2020). Evaluation of the Xpert MTB/RIF Test Accuracy for Diagnosis of Tuberculosis in Areas with a Moderate Tuberculosis burden. Apmis 129, 9–13. 10.1111/apm.13085 32975873

[B19] Nguyen T. B. Do D. N. Nguyen-Thanh T. Tatipamula V. B. Nguyen H. T. (2021). Identification of Five Hub Genes as Key Prognostic Biomarkers in Liver Cancer via Integrated Bioinformatics Analysis. Biology (Basel) 10 (10), 957. 10.3390/biology10100957 34681056 PMC8533228

[B20] Radanova M. Vasilev V. Deliyska B. Kishore U. Ikonomov V. Ivanova D. (2012). Anti-C1q Autoantibodies Specific against the Globular Domain of the C1qB-Chain from Patient with Lupus Nephritis Inhibit C1q Binding to IgG and CRP. Immunobiology 217 (7), 684–691. 10.1016/j.imbio.2011.11.007 22209113

[B21] Saxena V. Li L. Paluskievicz C. Kasinath V. Bean A. Abdi R. (2019). Role of Lymph Node Stroma and Microenvironment in T Cell Tolerance. Immunol. Rev. 292 (1), 9–23. 10.1111/imr.12799 31538349 PMC6935411

[B22] Singh S. K. Mishra M. K. Rivers B. M. Gordetsky J. B. Bae S. Singh R. (2020). Biological and Clinical Significance of the CCR5/CCL5 Axis in Hepatocellular Carcinoma. Cancers (Basel) 12 (4), 883. 10.3390/cancers12040883 PMC7226629 32260550

[B23] Smoot M. E. Ono K. Ruscheinski J. Wang P.-L. Ideker T. (2011). Cytoscape 2.8: New Features for Data Integration and Network Visualization. Bioinformatics 27 (3), 431–432. 10.1093/bioinformatics/btq675 21149340 PMC3031041

[B24] Szklarczyk D. Franceschini A. Wyder S. Forslund K. Heller D. Huerta-Cepas J. (2015). STRING V10: Protein-Protein Interaction Networks, Integrated over the Tree of Life. Nucleic Acids Res. 43, D447–D452. 10.1093/nar/gku1003 25352553 PMC4383874

[B25] Tavares L. P. Garcia C. C. Gonçalves A. P. F. Kraemer L. R. Melo E. M. Oliveira F. M. S. (2020). ACKR2 Contributes to Pulmonary Dysfunction by Shaping CCL5:CCR5-dependent Recruitment of Lymphocytes during Influenza A Infection in Mice. Am. J. Physiology-Lung Cell Mol. Physiol. 318 (4), L655–L670. 10.1152/ajplung.00134.2019 31995405

[B26] Wang L. Wen Z. Ma H. Wu L. Chen H. Zhu Y. (2021). Long Non-Coding RNAs ENST00000429730.1 and are Associated with Metabolic Activity in Tuberculosis Lesions of Sputum-Negative Tuberculosis Patients. Aging 13 (6), 8228–8247. 10.18632/aging.202634 33686954 PMC8034958

[B27] Wang T. Li W. Cheng H. Zhong L. Deng J. Ling S. (2019). The Important Role of the Chemokine Axis CCR7-CCL19 and CCR7-CCL21 in the Pathophysiology of the Immuno-Inflammatory Response in Dry Eye Disease. Ocul. Immunol. Inflamm. 29 (2), 266–277. 10.1080/09273948.2019.1674891 31702421

[B28] WHO (2019). Global Tuberculosis Report. World Health Organization; 2019. Available at: https://www.who.int/tb/global-report-2019 (Accessed 17 October 2019).

[B29] Xu H. Xing J. Tang X. Sheng X. Zhan W. (2020). The Effects of CCL3, CCL4, CCL19 and CCL21 as Molecular Adjuvants on the Immune Response to VAA DNA Vaccine in Flounder (*Paralichthys O*). Develop. Comp. Immunol. 103, 103492. 10.1016/j.dci.2019.103492 31494219

[B30] Xu M. Ouyang T. Lv K. Ma X. (2020). Integrated WGCNA and PPI Network to Screen Hub Genes Signatures for Infantile Hemangioma. Front. Genet. 11, 614195. 10.3389/fgene.2020.614195 33519918 PMC7844399

[B31] Yan Y. Chen R. Wang X. Hu K. Huang L. Lu M. (2019). CCL19 and CCR7 Expression, Signaling Pathways, and Adjuvant Functions in Viral Infection and Prevention. Front. Cel Dev. Biol. 7, 212. 10.3389/fcell.2019.00212 PMC6781769 31632965

[B32] Ye C. Zhu S. Yuan J. (2021). Construction of ceRNA Network to Reveal Potential Biomarkers in Crohn's Disease and Validation in a TNBS Induced Mice Model. J. Inflamm. Res. 14, 6447–6459. 10.2147/jir.S338053 34880646 PMC8648272

[B33] Yu-Ju Wu C. Chen C.-H. Lin C.-Y. Feng L.-Y. Lin Y.-C. Wei K.-C. (2020). CCL5 of Glioma-Associated Microglia/Macrophages Regulates Glioma Migration and Invasion via Calcium-Dependent Matrix Metalloproteinase 2. Neuro-oncology 22 (2), 253–266. 10.1093/neuonc/noz189 31593589 PMC7032635

[B34] Zhao R. Qi S. Cui Y. Gao Y. Jiang S. Zhao J. (2022). Transcriptomic and Physiological Analysis Identifies a Gene Network Module Highly Associated with Brassinosteroid Regulation in Hybrid Sweetgum Tissues Differing in the Capability of Somatic Embryogenesis. Hortic. Res. 9, uhab047. 10.1093/hr/uhab047 35031801 PMC8788368

[B35] Zhao Y. Ma T. Zou D. (2021). Identification of Unique Transcriptomic Signatures and Hub Genes through RNA Sequencing and Integrated WGCNA and PPI Network Analysis in Nonerosive Reflux Disease. J. Inflamm. Res. 14, 6143–6156. 10.2147/jir.S340452 34848992 PMC8627320

[B36] Zhou G. Soufan O. Ewald J. Hancock R. E. W. Basu N. Xia J. (2019). NetworkAnalyst 3.0: A Visual Analytics Platform for Comprehensive Gene Expression Profiling and Meta-Analysis. Nucleic Acids Res. 47, W234–W241. 10.1093/nar/gkz240 30931480 PMC6602507

[B37] Zhou Y. Zhou B. Pache L. Chang M. Khodabakhshi A. H. Tanaseichuk O. (2019). Metascape Provides a Biologist-Oriented Resource for the Analysis of Systems-Level Datasets. Nat. Commun. 10 (1), 1523. 10.1038/s41467-019-09234-6 30944313 PMC6447622

[B38] Zimmer A. J. Lainati F. Aguilera Vasquez N. Chedid C. McGrath S. Benedetti A. (2021). Biomarkers that Correlate with Active Pulmonary Tuberculosis Treatment Response: A Systematic Review and Meta-Analysis. J. Clin. Microbiol., JCM0185921. 10.1128/jcm.01859-21 PMC8849205 34911364

[B39] Zuo Z. Shen J.-X. Pan Y. Pu J. Li Y.-G. Shao X.-h. (2018). Weighted Gene Correlation Network Analysis (WGCNA) Detected Loss of MAGI2 Promotes Chronic Kidney Disease (CKD) by Podocyte Damage. Cell Physiol Biochem 51 (1), 244–261. 10.1159/000495205 30448842

